# Polymerization-induced self-assembly and disassembly during the synthesis of thermoresponsive ABC triblock copolymer nano-objects in aqueous solution†

**DOI:** 10.1039/d2sc01611g

**Published:** 2022-06-08

**Authors:** Spyridon Varlas, Thomas J. Neal, Steven P. Armes

**Affiliations:** Department of Chemistry, University of Sheffield Dainton Building, Brook Hill Sheffield S3 7HF UK s.varlas@sheffield.ac.uk s.p.armes@sheffield.ac.uk

## Abstract

Polymerization-induced self-assembly (PISA) has been widely utilized as a powerful methodology for the preparation of various self-assembled AB diblock copolymer nano-objects in aqueous media. Moreover, it is well-documented that chain extension of AB diblock copolymer vesicles using a range of *hydrophobic* monomers *via* seeded RAFT aqueous emulsion polymerization produces *framboidal* ABC triblock copolymer vesicles with adjustable surface roughness owing to microphase separation between the two enthalpically incompatible hydrophobic blocks located within their membranes. However, the utilization of *hydrophilic* monomers for the chain extension of linear diblock copolymer vesicles has yet to be thoroughly explored; this omission is addressed for aqueous PISA formulations in the present study. Herein poly(glycerol monomethacrylate)-poly(2-hydroxypropyl methacrylate) (G-H) vesicles were used as seeds for the RAFT aqueous dispersion polymerization of oligo(ethylene glycol) methyl ether methacrylate (OEGMA). Interestingly, this led to *polymerization-induced disassembly* (PIDA), with the initial precursor vesicles being converted into lower-order worms or spheres depending on the target mean degree of polymerization (DP) for the corona-forming POEGMA block. Moreover, construction of a pseudo-phase diagram revealed an unexpected copolymer concentration dependence for this PIDA formulation. Previously, we reported that PHPMA-based diblock copolymer nano-objects only exhibit thermoresponsive behavior over a relatively narrow range of compositions and DPs (see Warren *et al.*, *Macromolecules*, 2018, **51**, 8357–8371). However, introduction of the POEGMA coronal block produced thermoresponsive ABC triblock nano-objects even when the precursor G-H diblock copolymer vesicles proved to be thermally unresponsive. Thus, this new approach is expected to enable the rational design of new nano-objects with tunable composition, copolymer architectures and stimulus-responsive behavior.

## Abstract 2

Chain extension of linear AB diblock copolymer vesicles by seeded RAFT aqueous dispersion polymerization using a hydrophilic monomer C leads to *polymerization-induced disassembly* to form lower-order thermoresponsive ABC triblock copolymer nano-objects.

## Graphical abstract

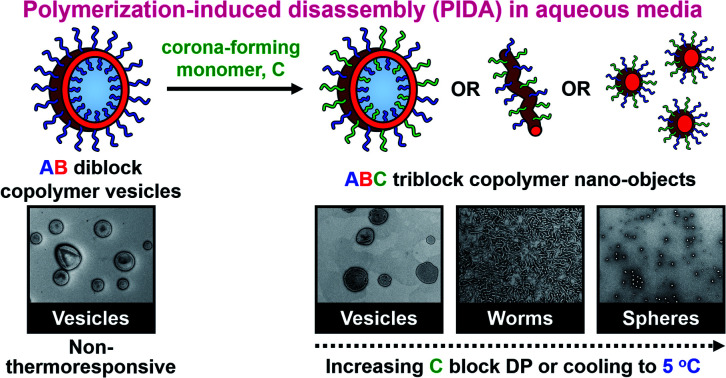

## Introduction

Block copolymer vesicles (or polymersomes) are self-assembled nanostructures that possess an inner aqueous cavity enclosed by a hydrophobic membrane.^1–4^ Typically, vesicles are prepared by self-assembly of amphiphilic AB diblock copolymers in aqueous media at copolymer concentrations of no more than 1% w/w using either solvent-switch or thin-film rehydration.^5–8^ The ability of such vesicles to simultaneously deliver hydrophilic and hydrophobic payloads suggests their utilization for various potential biomedical applications, including drug/gene delivery,^9–11^ cell/organelle mimicry,^12–15^ diagnostic imaging^16,17^ and sensors.^18,19^

Over the past decade or so, polymerization-induced self-assembly (PISA) has emerged as a versatile platform technology for the efficient synthesis and self-assembly of amphiphilic AB diblock copolymers in a single step to produce sterically-stabilized nanoparticles at up to 50% w/w solids.^20–26^ Systematic adjustment of the target diblock copolymer compositions enables the reproducible targeting of well-defined spheres, worms or vesicles.^27–29^ In particular, block copolymer vesicles prepared *via* aqueous PISA formulations have attracted significant interest owing to their promise as drug/protein delivery vehicles,^30–33^ cell-mimicking nanoreactors,^34–37^ Pickering emulsifiers^38,39^ ice recrystallization inhibitors^40,41^ and bioimaging tools.^42,43^

Recently, PISA formulations have been also used to prepare triblock copolymer nano-objects.^44–51^ In many cases, AB diblock copolymer vesicles are first synthesized *via* aqueous dispersion polymerization and subsequently used as ‘seed’ particles for chain extension *via* aqueous emulsion polymerization using various *hydrophobic* monomers. This results in the formation of framboidal ABC triblock copolymer vesicles of tunable surface roughness.^52–54^ This is attributable to the enthalpic incompatibility between the core-forming B and C blocks that, in turn, leads to microphase separation within the vesicular membrane. Such model particles have been evaluated as next-generation Pickering emulsifiers since they exhibit superior adsorption efficiency compared to conventional vesicles.^55^ Optimization of their surface roughness also facilitates intracellular uptake of framboidal vesicles owing to their similarity to the infectious form of the Dengue virus.^56^

One of the most widely employed vinyl monomers used for the preparation of block copolymer nano-objects *via* aqueous PISA is 2-hydroxypropyl methacrylate (HPMA).^28,57–59^ Interestingly, PHPMA-based diblock copolymer nano-objects are weakly thermosensitive, which leads to shape-shifting behavior when adjusting the solution temperature.^60–62^ For example, poly(glycerol monomethacrylate)-poly(2-hydroxypropyl methacrylate) (G-H) diblock copolymer worms or vesicles can be transformed into spheres on cooling from ambient temperature to either 4 °C or 0 °C, respectively.^63,64^ Notably, an aqueous dispersion of a single PHPMA-based diblock copolymer has been demonstrated to form spheres, worms or vesicles simply by adjusting the solution temperature.^65^ However, such thermoresponsive behavior can suffer from hysteresis and only occurs over a relatively narrow range of copolymer compositions. For example, it is well-established that a relatively high degree of polymerization (DP) leads to PHPMA chains that are no longer thermoresponsive.^66^

Herein we investigate the chain extension of G-H diblock copolymer vesicles by seeded reversible addition–fragmentation chain transfer (RAFT) aqueous dispersion polymerization using a *hydrophilic* monomer (oligo(ethylene glycol) methyl ether methacrylate; OEGMA or O). Such formulations produce asymmetric ABC triblock copolymer nano-objects (G-H-O) in which both the A and C block are hydrophilic, which has profound consequences for the copolymer morphology. Moreover, the core-forming B block comprised relatively long PHPMA chains, which were sufficiently hydrophobic to ensure that the G-H precursor vesicles did not exhibit any thermoresponsive character. The following questions were addressed. How does the copolymer morphology evolve as the target DP for the POEGMA block and the total solids concentration are systematically varied? How does the copolymer morphology evolve during the polymerization when targeting a relatively high POEGMA DP? Does the introduction of a hydrophilic C block have any influence on the (non)-thermoresponsive behavior of the PHPMA chains? Our findings extend our understanding of thermoresponsive block copolymer nano-objects and establish new synthesis-structure–property relationships that are expected to inform their rational design.

## Results and discussion

First, a poly(glycerol monomethacrylate)_59_ (G_59_) macromolecular chain transfer agent (macro-CTA) was synthesised *via* RAFT solution polymerization of glycerol monomethacrylate (GMA) in anhydrous ethanol at 70 °C using a carboxylic acid-functionalized dithiobenzoate CTA and 4,4′-azobis(4-cyanovaleric acid) (ACVA) as the radical initiator. After 180 min, a final GMA conversion of 85% was determined by ^1^H NMR analysis in CD_3_OD and a mean DP of 59 was also determined by end-group analysis of the purified G_59_ precursor. Size exclusion chromatography (SEC) analysis using DMF eluent containing 10 mM LiBr revealed a narrow unimodal molecular weight distribution with an *M*_n_ of 16.2 kg mol^−1^ and a dispersity (*M*_w_/*M*_n_) of 1.14 (Fig. S1†). Such data indicate good control for this RAFT solution polymerization.

**Scheme 1:**
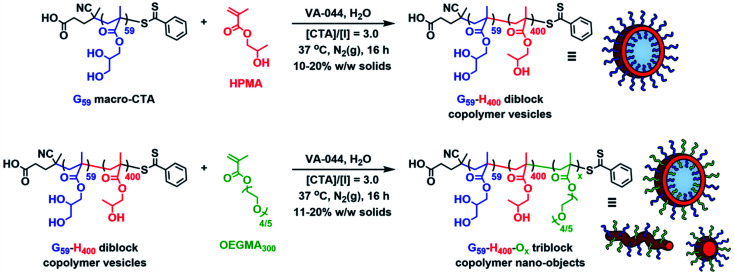
Schematic illustration of the synthesis route employed for the preparation of poly(glycerol monomethacrylate)_59_-poly(2-hydroxypropyl methacrylate)_400_ (G_59_-H_400_) diblock copolymer vesicles at 10–20% w/w solids *via* RAFT aqueous dispersion polymerization of HPMA using a G_59_ precursor, and their subsequent utilization as seed particles for the preparation of G_59_-H_400_-O_*x*_ triblock copolymer nano-objects at 11–20% w/w solids *via* RAFT-mediated aqueous *polymerization-induced disassembly* (PIDA).

This water-soluble G_59_ precursor was subsequently chain-extended *via* RAFT aqueous dispersion polymerization of 2-hydroxypropyl methacrylate (HPMA) at 37 °C using 2,2′-azobis[2-(2-imidazolin-2-yl)propane]dihydrochloride (VA-044) as the radical initiator to produce G_59_-H_400_ diblock copolymer vesicles at 10%, 15% or 20% w/w solids (Scheme 1).^67^ In each case, more than 99% HPMA conversion was achieved within 16 h, as determined by ^1^H NMR analysis in CD_3_OD. SEC analysis using DMF eluent containing 10 mM LiBr indicated efficient chain extension and relatively narrow molecular weight distributions (*M*_w_/*M*_n_ ≤ 1.15), with no detectable amount of any unreacted G_59_ precursor (Fig. S2 and Table S1†).

Dynamic light scattering (DLS) analysis revealed monomodal particle size distributions for these G_59_-H_400_ diblock copolymer vesicles, with mean hydrodynamic diameters (*D*_h_) ranging from 350 to 450 nm and relatively low DLS polydispersities (PD ≤ 0.20) (Fig. S3A and Table S1†). Moreover, aqueous electrophoresis studies confirmed their strongly anionic character with zeta potentials ranging between −28 and −30 mV at pH 6.8 (which is the pH of deionized water used for the PISA and PIDA syntheses and subsequent characterization), owing to anionic carboxylate end-groups on the steric stabilizer chains expressed at the vesicle outer surface (Table S1†).

**Fig. 1:**
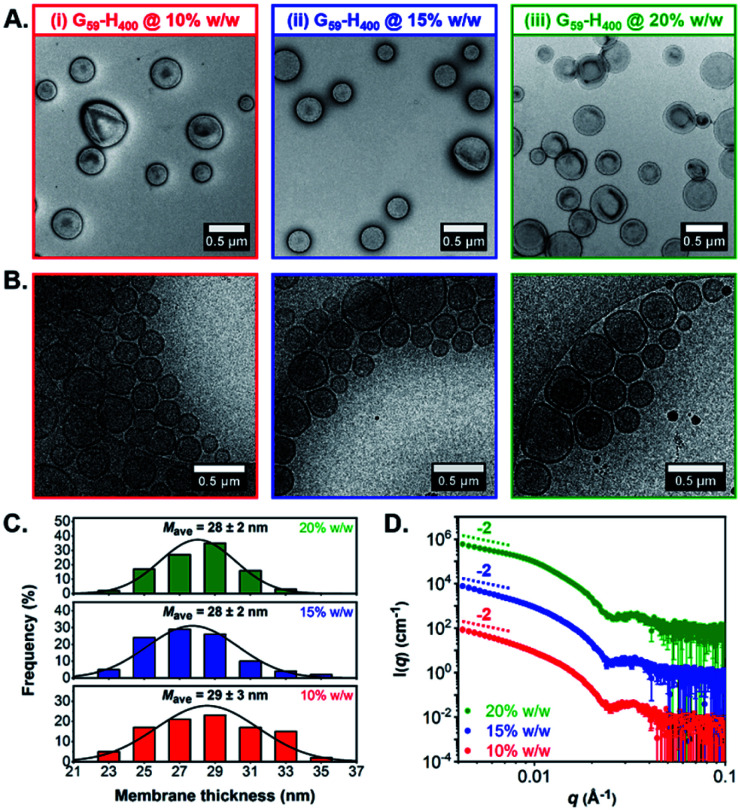
Characterization of G_59_-H_400_ diblock copolymer precursor vesicles prepared *via* RAFT-mediated aqueous PISA when targeting (i) 10% w/w, (ii) 15% w/w or (iii) 20% w/w solids: (A) representative dry-state TEM images obtained using a 0.75% w/w uranyl formate staining solution; (B) corresponding representative cryo-TEM images; (C) membrane thickness distribution histograms and mean membrane thicknesses, determined by analyzing at least 100 vesicles in each case; (D) SAXS patterns recorded for 1.0% w/w aqueous vesicle dispersions.

Both conventional and cryogenic transmission electron microscopy (TEM) analysis revealed the formation of unilamellar G_59_-H_400_ diblock copolymer vesicles for aqueous PISA syntheses conducted at 10%, 15% or 20% w/w solids (Fig. 1A, B, S3B and C†). Analysis of the cryo-TEM images also enabled calculation of the mean membrane thickness (*M*_ave_), which was estimated to be approximately 28–29 nm in each case (Fig. 1C). Moreover, small-angle X-ray scattering (SAXS) analysis was consistent with the DLS and TEM data. The vesicle morphology was confirmed by the characteristic −2 gradient in the low-*q* region (where *q* is the scattering vector) of the recorded patterns and the local minima observed at *q* ≈ 0.025 Å^−1^ enabled mean vesicle membrane thicknesses of 24–25 nm to be calculated, using *d* = 2π/*q* (where *d* is a real-space distance in Å and *q* is the local minimum at ∼0.025 Å^−1^) (Fig. 1D).^68^

**Fig. 2:**
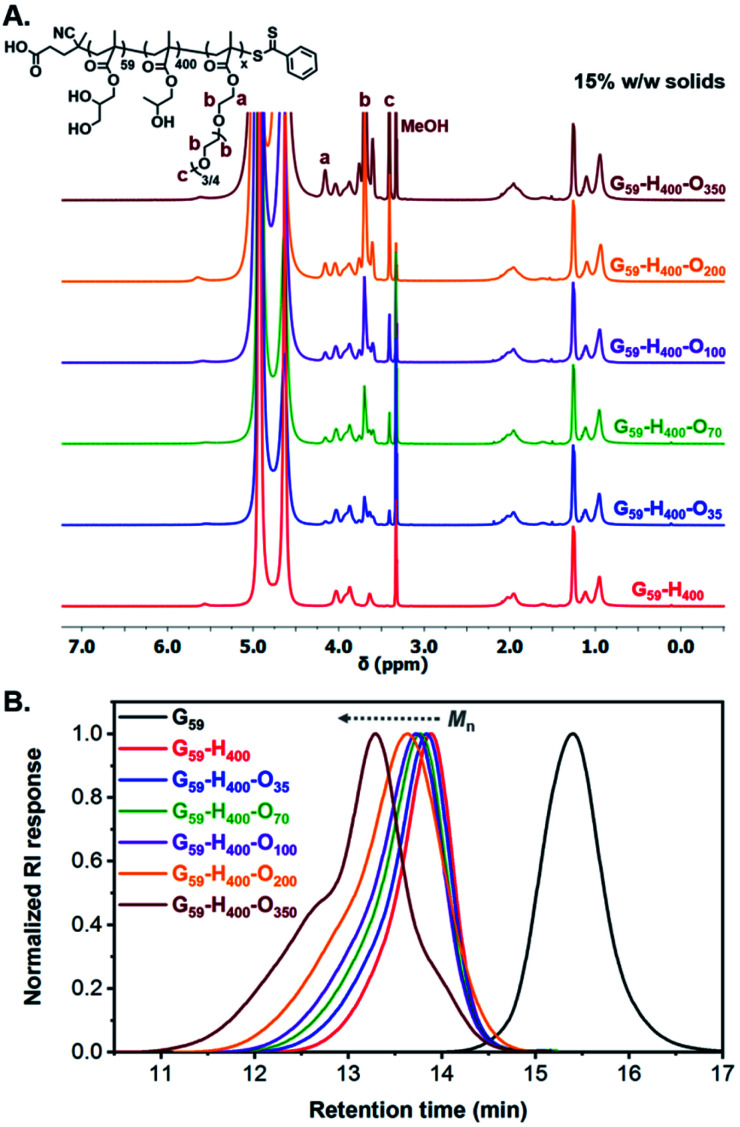
(A) Stacked ^1^H NMR spectra (CD_3_OD) recorded for a series of molecularly-dissolved G_59_-H_400_-O_*x*_ triblock copolymers (where *x* = 35, 70, 100, 200 or 350) prepared *via* RAFT-mediated aqueous PIDA at 15% w/w solids. (B) Normalized SEC traces (refractive index detector) recorded for the G_59_ precursor (black line), the G_59_-H_400_ diblock copolymer (red line) and a series of G_59_-H_400_-O_*x*_ triblock copolymers (where *x* = 35, blue line; *x* = 70, green line; *x* = 100, purple line; *x* = 200, orange line; *x* = 350, burgundy line) at 15% w/w solids (DMF + 10 mM LiBr eluent).

In principle, utilization of these G_59_-H_400_ diblock copolymer vesicles as seed particles for chain extension experiments using a hydrophilic corona-forming monomer should lead to their *polymerization-induced disassembly* (PIDA) to form ABC triblock copolymer nano-objects with lower-order morphologies. To examine this hypothesis, the RAFT polymerization of hydrophilic oligo(ethylene glycol) methyl ether methacrylate (OEGMA; average *M*_n_ = 300) was conducted in the presence of G_59_-H_400_ seed vesicles at 37 °C using VA-044 as the radical initiator (Scheme 1). A series of G_59_-H_400_-O_*x*_ triblock copolymer nano-objects were produced when targeting *x* = 35, 70, 100, 200 or 350 at 11%, 15% or 20% w/w solids. ^1^H NMR analysis (CD_3_OD) of the molecularly-dissolved G_59_-H_400_-O_*x*_ triblock copolymers indicated that more than 95% OEGMA conversion was achieved within 16 h in all cases, with characteristic new signals for the corona-forming POEGMA block being observed at 3.4–4.2 ppm (Fig. 2A, S4 and S5 and Table S1†).

Notably, the resulting G_59_-H_400_-O_*x*_ triblock copolymers possessed unimodal molecular weight distributions and *M*_n_ values that increased linearly when targeting higher POEGMA DPs, as determined by SEC analysis using DMF eluent. A high molecular weight shoulder was observed for both G_59_-H_400_-O_200_ and G_59_-H_400_-O_350_, possibly owing to the greater probability of termination by combination when targeting a relatively high DP for the third block. Alternatively, this SEC feature may indicate that some degree of chain transfer to polymer occurs during such syntheses. As typically observed for block copolymers prepared *via* PISA, higher dispersities were observed when targeting (i) longer corona-forming POEGMA blocks or (ii) higher solids concentrations for compositionally identical triblock copolymers (Fig. 2B, S6 and S7 and Table S1†).^69^ In all cases, the SEC traces clearly shift toward higher retention times. Moreover, the UV traces (*λ* = 305 nm, which corresponds to the absorption maximum for the dithiobenzoate end-group) overlap with the corresponding refractive index traces. This confirms the effective partitioning of OEGMA within the hydrophobic PHPMA-based membranes of the seed diblock copolymer vesicles during aqueous PIDA and its controlled RAFT polymerization, rather than its uncontrolled free radical polymerization in the aqueous continuous phase.

**Fig. 3:**
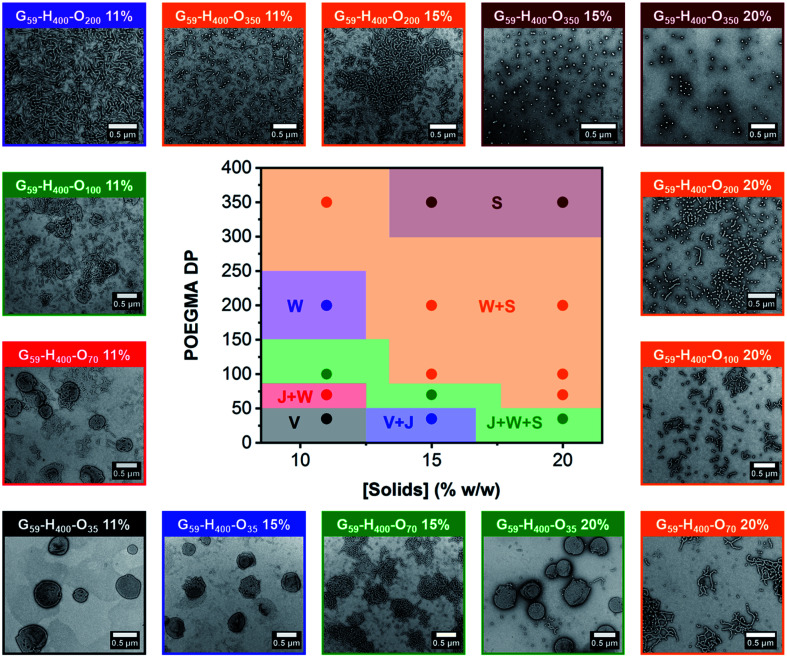
Pseudo-phase diagram constructed for G_59_-H_400_-O_*x*_ triblock copolymer nano-objects (*x* = 35–350) prepared *via* RAFT-mediated aqueous PIDA by systematically varying the total solids content and target POEGMA DP. Representative TEM images obtained using a 0.75% w/w uranyl formate staining solution are shown for each formulation. Each frame color corresponds to a different nano-object morphology (S = spherical micelles; W = worms; J = jellyfish and V = vesicles).

The resulting series of G_59_-H_400_-O_*x*_ triblock copolymer nano-objects were characterized by DLS, conventional TEM and aqueous electrophoresis to examine (i) their morphological evolution and (ii) any change in their electrophoretic footprint on introducing a second non-ionic coronal block (*i.e.*, POEGMA). In general, increasing either the target POEGMA DP (for nano-objects prepared *via* aqueous PIDA at the same solids content) or the copolymer concentration (for nano-objects prepared with identical POEGMA block DPs) resulted in the formation of lower-order morphologies, such as worms (W) and spheres (S). These findings were corroborated by constructing a pseudo-phase diagram (Fig. 3 and S8†). In some cases, mixed morphologies were obtained and transient species such as jellyfish (J) were also observed. This change in copolymer morphology complements the evolution from spheres to worms to vesicles reported during prototypical aqueous PISA formulations when targeting block copolymer vesicles.^27,68,70^ For example, perforated vesicles (V; containing one or more point defects), a mixture of vesicles and jellyfish (V + J) or a mixture of jellyfish, worms and spheres (J + W + S) were formed by G_59_-H_400_-O_35_ nano-objects prepared at 11%, 15% or 20% w/w solids, respectively. Moreover, intermediate morphologies, such as jellyfish and worms (J + W) or worms and spheres (W + S) were observed between pure copolymer morphologies. Importantly, a pure phase comprising branched worms was obtained when targeting G_59_-H_400_-O_200_ at 11% w/w solids, while sub-100 nm spherical micelles were obtained when targeting G_59_-H_400_-O_350_ at either 15% or 20% w/w solids. As expected, mixtures of nano-object morphologies generally exhibited higher DLS polydispersities compared to the corresponding pure phases (Table S1†). The formation of lower-order morphologies by G_59_-H_400_-O_*x*_ triblock copolymer nano-objects was also supported by visual inspection of these aqueous dispersions. For example, when targeting G_59_-H_400_-O_*x*_*via* aqueous PIDA at 20% w/w solids, the initial turbid, free-flowing aqueous dispersion of G_59_-H_400_ vesicles was converted into either an opaque or a transparent free-standing gel (depending on the worm fraction) or a transparent, free-flowing dispersion comprising non-interacting G_59_-H_400_-O_350_ spheres. For the range of POEGMA DPs investigated, it is emphasized that there was no evidence for the formation of molecularly-dissolved triblock copolymer chains. This is attributed to the hydrophobic nature of the PHPMA block, which prevents complete dissociation to individual copolymer chains.^54^

Moreover, synthesis of a series of G_59_-H_400_-O_*x*_ triblock copolymer nano-objects *via* aqueous PIDA also led to discernible changes in their electrophoretic behavior relative to that of the original G_59_-H_400_ diblock copolymer vesicles. In particular, targeting longer non-ionic POEGMA blocks led to the highly negative zeta potentials observed for the initial seed vesicles (−28 to −30 mV) being drastically reduced to near-neutral values ranging from −3.9 to −11 mV for G_59_-H_400_-O_200_ or G_59_-H_400_-O_350_ triblock copolymer nano-objects prepared at 11–20% w/w solids (Fig. S9 and Table S1†). For the shortest third block targeted in this copolymer series (POEGMA DP = 35), this is attributed to dilution of the steric stabilizer layer with non-ionic POEGMA chains. For the longer POEGMA blocks, charge screening of the anionic carboxylate end-groups on the G_59_ chains is expected to occur as these new steric stabilizer chains exceed the DP of the original corona-forming block.

To monitor the evolution in copolymer morphology during RAFT-mediated aqueous PIDA (and also assess the extent of control achieved during the OEGMA polymerization), a kinetic study was performed during the synthesis of G_59_-H_400_-O_350_ triblock copolymer spheres at 37 °C when targeting 15% w/w solids. Thus, 200 μL aliquots were periodically withdrawn from the polymerizing mixture every 30 min for 4 h for subsequent analysis by ^1^H NMR spectroscopy (CD_3_OD), SEC using DMF eluent and conventional TEM. This particular PIDA formulation was selected to enable monitoring of the *in situ* evolution in morphology from triblock copolymer vesicles to worms to spheres, while simultaneously allowing assessment of the degree of RAFT control achieved when targeting the highest POEGMA DP.

**Fig. 4:**
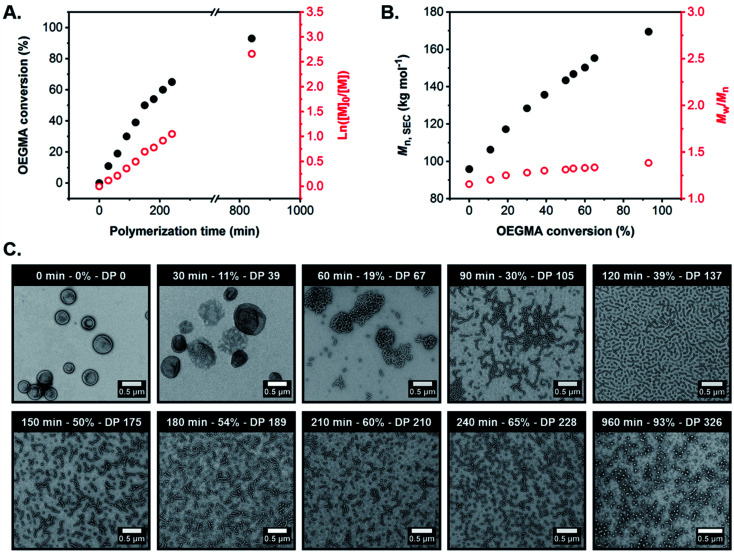
(A) OEGMA conversion *vs.* time curve (black circles) and corresponding ln([*M*]_0_/[*M*]) *vs.* time plot (red circles) determined by ^1^H NMR spectroscopy studies (CD_3_OD) during the synthesis of G_59_-H_400_-O_350_ triblock copolymer spheres *via* RAFT-mediated aqueous PIDA at 37 °C targeting 15% w/w solids. (B) Evolution in *M*_n_ (black circles) and *M*_w_/*M*_n_ (red circles) with OEGMA conversion for a series of G_59_-H_400_-O_*x*_ triblock copolymers prepared at 15% w/w solids, as calculated from SEC analysis (refractive index detector) using a series of PMMA calibration standards (DMF eluent + 10 mM LiBr). (C) Representative TEM images recorded for G_59_-H_400_-O_*x*_ triblock copolymer nano-objects formed during the synthesis of G_59_-H_400_-O_350_ triblock copolymer spheres *via* RAFT-mediated aqueous PIDA at 37 °C targeting 15% w/w solids, obtained using a uranyl formate stain. The polymerization time, OEGMA conversion and corresponding POEGMA DP are indicated for each image.

According to the conversion *vs.* time curve and corresponding semi-logarithmic plot, the OEGMA polymerization exhibited pseudo-first order kinetics with no apparent change in the rate of polymerization up to 93% monomer conversion (POEGMA DP = 326), which was achieved after 16 h at 37 °C (Fig. 4A, and S10 and Table S2†). Furthermore, SEC analysis of the withdrawn aliquots confirmed the synthesis of triblock copolymers with unimodal molecular weight distributions, relatively low dispersities (*M*_w_/*M*_n_ ≤ 1.38) and *M*_n_ values that increased linearly with OEGMA conversion, suggesting that a well-controlled RAFT polymerization occurred under these conditions (Fig. 4B and S11 and Table S2†).

TEM analysis of the G_59_-H_400_-O_*x*_ triblock copolymer nano-objects present at various OEGMA conversions and mean POEGMA DPs indicated a morphological evolution from vesicles to worms to spheres, in good agreement with the *post-mortem* morphologies observed at full OEGMA conversion when targeting similar POEGMA DPs (Fig. 4C and S12†). This suggests that the evolving copolymer morphologies observed during the OEGMA polymerization were not affected by the presence of unreacted monomer. Specifically, TEM analysis suggested that the original G_59_-H_400_ seed vesicles initially unwrap *via* perforated membranes to form jellyfish at 11% OEGMA conversion (30 min; G_59_-H_400_-O_39_). These intermediate structures then undergo further disassembly to form interconnected networks comprising highly branched worms at 19% conversion (60 min; G_59_-H_400_-O_67_). Higher OEGMA conversions (30–65%, 90–240 min; G_59_-H_400_-O_105-228_) resulted in the formation of progressively shorter worms with minimal branching and the concurrent appearance of a population of spheres, arising from the synthesis of highly asymmetric amphiphilic triblock copolymers. Ultimately, the remaining population of short worms was transformed into an almost pure phase of sub-100 nm spheres at the highest OEGMA conversion (93%, 960 min; G_59_-H_400_-O_326_).

Previously, we reported that PHPMA-based diblock copolymer nano-objects exhibit thermoresponsive behavior.^63–66^ Thus, an aqueous dispersion of G_54_-H_140_ worms can be converted into spheres on cooling from 21 °C to 4 °C.^63^ Similarly, G_58_-H_250_ vesicles can be transformed into spheres by cooling to 0 °C.^64^ More recently, we demonstrated that a single PHPMA-based diblock copolymer could form spheres, worms or vesicles in aqueous media simply by adjusting the solution temperature.^65^ However, such thermoresponsive behavior often suffers from hysteresis^65^ and is typically only observed over a rather narrow range of block copolymer compositions.^66^ Consequently, we hypothesized that our aqueous PIDA approach to the synthesis of ABC triblock copolymer nano-objects, which produces hydrophilic blocks on either side of the core-forming PHPMA block, might lead to thermoresponsive character over a significantly wider range of target PHPMA DPs than those reported for G-H nano-objects.^66,71^

**Fig. 5:**
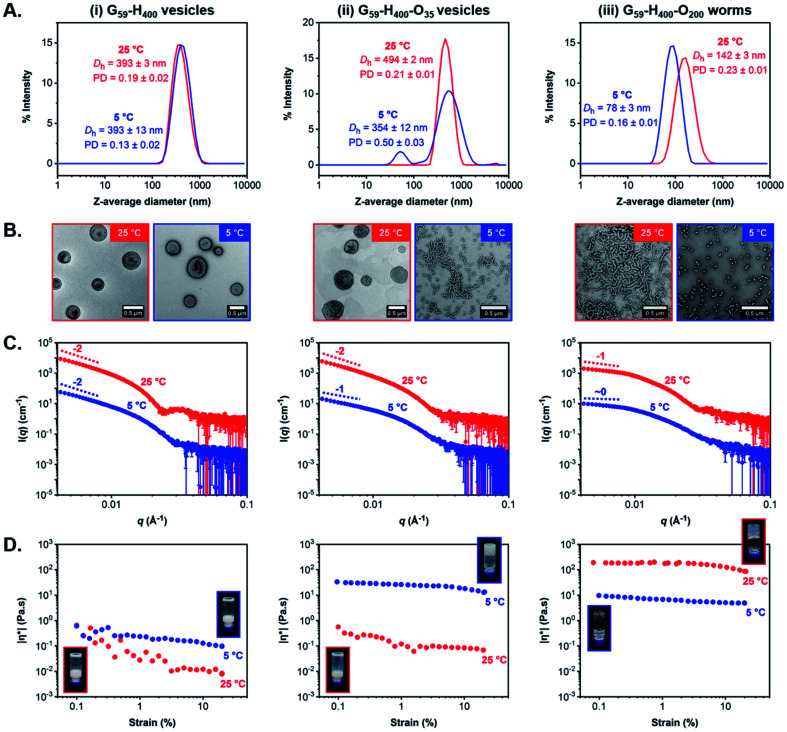
Characterization data obtained for (i) G_59_-H_400_ diblock copolymer vesicles, (ii) G_59_-H_400_-O_35_ triblock copolymer nano-objects, and (iii) G_59_-H_400_-O_200_ triblock copolymer nano-objects at 25 °C (red data) and 5 °C (blue data) after incubation at each temperature for 24 h prior to analysis: (A) intensity-weighted particle size distributions, mean *D*_h_ values and polydispersities determined by DLS analysis of 0.1% w/w aqueous dispersions (the error indicates the standard deviation from at least three repeat measurements). (B) Representative TEM images obtained using a uranyl formate stain. (C) SAXS patterns recorded for 1.0% w/w aqueous dispersions. (D) Complex viscosity (|η*|) as a function of applied strain (insets show digital images recorded for the corresponding aqueous copolymer dispersions at 10–11% w/w solids, which indicate whether they form a free-standing gel or a free-flowing fluid in each case).

First, we verified that the initial G_59_-H_400_ diblock copolymer vesicles prepared *via* aqueous PISA at 10% w/w solids were not thermoresponsive when cooled from 25 °C to 5 °C for 24 h. Indeed, DLS and TEM analysis indicated the presence of well-defined unilamellar vesicles of identical size (*D*_h_ = 393 nm and PD ≤ 0.19) at both temperatures (Fig. 5A–i and B-i and S13†). Similarly, variable-temperature SAXS analysis also confirmed that the original vesicular morphology was retained on cooling from 25 °C to 5 °C, as judged by the low-*q* gradient of −2 observed for both patterns, although a shift in the local minima from *q* = 0.025 Å^−1^ at 25 °C to *q* = 0.030 Å^−1^ at 5 °C suggested a modest reduction in the mean vesicle membrane thickness from 25 nm to 21 nm (Fig. 5C–i and S14A†). Moreover, rheology studies conducted on a 10% w/w aqueous dispersion of G_59_-H_400_ vesicles as a function of applied strain demonstrated a free-flowing fluid of low complex viscosity (|η*|) at both 25 °C and 5 °C (Fig. 5D–i and S15A†).

To facilitate direct comparison, G_59_-H_400_-O_35_ triblock copolymer vesicles and G_59_-H_400_-O_200_ triblock copolymer worms prepared *via* aqueous PIDA at 11% w/w solids were characterized using the same analytical techniques to investigate their thermoresponsive behavior on cooling from 25 °C to 5 °C for 24 h. Indeed, the membrane-perforated G_59_-H_400_-O_35_ vesicles with a *D*_h_ of approximately 500 nm (PD = 0.21) originally formed at 25 °C were transformed into a pure phase comprising branched worms with *D*_h_ = 354 nm and PD = 0.50 on cooling to 5 °C, as indicated by TEM and DLS analysis (Fig. 5A-ii, B-ii and S13†). This morphological transition was also observed in the corresponding SAXS patterns recorded for the same formulation following incubation at either temperature for 24 h. More specifically, a characteristic change in the low-*q* gradient from −2 to −1 occurred on cooling from 25 °C to 5 °C, which is consistent with the formation of highly anisotropic worms in the latter case (Fig. 5C-ii and S14B†).^60,65^ Moreover, this thermally-induced morphological transition was accompanied by a macroscopic sol–gel transition to produce a soft, free-standing worm gel at 11% w/w solids. This accounts for the significantly higher |η*| compared to that recorded for the turbid, free-flowing G_59_-H_400_-O_35_ vesicle dispersion at 25 °C (Fig. 5D-ii and S15B†).

In striking contrast, the highly anisotropic G_59_-H_400_-O_200_ triblock copolymer worms (*D*_h_ = 142 nm and PD = 0.23) formed at 25 °C when targeting 11% w/w solids were transformed into spherical micelles (*D*_h_ = 78 nm and PD = 0.16) on cooling to 5 °C (Fig. 5A-iii, B-iii and S13†). TEM studies confirmed a predominantly spherical morphology, but also indicated the presence of a minor population of short worms. This thermally-induced worm-to-sphere transition was verified by SAXS analysis, since the initial low-*q* gradient of −1 observed for the worms at 25 °C was reduced to almost zero for the spheres formed at 5 °C (Fig. 5C-iii and S14C†). Furthermore, the G_59_-H_400_-O_200_ triblock copolymer worms formed a transparent, free-standing gel at 25 °C, but a gel–sol transition occurred on cooling to 5 °C to produce a transparent, free-flowing dispersion of spheres with a markedly lower complex viscosity (Fig. 5D-iii and S15C†). Interestingly, such shape-shifting thermoresponsive behavior was observed for both relatively short and significantly longer coronal POEGMA blocks. However, in each case the thermally-induced change in copolymer morphology proved to be irreversible over an experimental time scale of approximately one week. Nevertheless, it is worth emphasizing that the DP of the hydrophobic PHPMA block is approximately double that reported for poorly thermoresponsive vesicles by Lovett and co-workers^71^ and thermoresponsive worms by Warren and co-workers.^66^ This serves to highlight the importance and versatility of our new PIDA approach for the rational design of next-generation stimulus-responsive block copolymer formulations with tunable composition, architecture and morphology.

## Conclusions

In summary, *polymerization-induced disassembly* (PIDA) enables the preparation of ABC triblock copolymer nano-objects (where A and C represent the corona-forming blocks and B represents the core-forming block) with tunable compositions and morphologies in aqueous media. First, AB diblock copolymer vesicles (*i.e.*, G_59_-H_400_) are prepared *via* RAFT aqueous dispersion polymerization and subsequently used as seeds for a series of chain extension experiments using a second water-soluble monomer (*i.e.*, OEGMA). This results in the formation of asymmetric ABC triblock copolymer nano-objects (*i.e.*, G_59_-H_400_-O_*x*_) with a progressive evolution in morphology toward lower-order nanostructures, such as jellyfish, worms or spheres when targeting longer C blocks or increasing the copolymer concentration. Kinetic studies conducted during aqueous PIDA provide useful mechanistic insights regarding the observed morphological transitions. Importantly, the final ABC triblock copolymer nano-objects exhibit thermoresponsive behavior, which is not observed for the original AB diblock copolymer vesicles. More specifically, vesicles are transformed into worms, while worms can be converted into spheres on cooling from ambient temperature to 5 °C. Overall, this study sheds important new light on the self-assembly of amphiphilic diblock and triblock copolymers in aqueous solution and is expected to inform the design of next-generation stimulus-responsive block copolymer nano-objects.

## Data availability

Relevant additional data are provided in the ESI.†

## Author contributions

S. V. and S. P. A. conceived this study. S. V. performed all the experiments and analyzed the data. S. P. A. acquired the funding for this research project. S. V. and S. P. A. wrote and edited the manuscript. T. J. N. performed the SAXS and rheology experiments and analyzed the resulting data.

## Conflicts of interest

There are no conflicts to declare.

## Supplementary Material

SC-013-D2SC01611G-s001
