# Supplementary material for: Polymerization-induced self-assembly and disassembly during the synthesis of thermoresponsive ABC triblock copolymer nano-objects in aqueous solution
Source: Chem Sci. 2022 Jun 8;13(24):7295–303. doi: 10.1039/d2sc01611g (PMC9214878; doi:10.1039/d2sc01611g)
Supplement: SC-013-D2SC01611G-s001 [file SC-013-D2SC01611G-s001.pdf]

Electronic Supplementary Material (ESI) for Chemical Science.
This journal is © The Royal Society of Chemistry 2022

Electronic Supplementary Information for:

Polymerization-induced self-assembly and disassembly during the synthesis of thermoresponsive ABC triblock copolymer nano-objects in aqueous solution

Spyridon Varlas, Thomas J. Neal, and Steven P. Armes**

Department of Chemistry, University of Sheffield, Dainton Building, Brook Hill, Sheffield, S3 7HF, UK

*Corresponding Authors: s.varlas@sheffield.ac.uk (S.V.), s.p.armes@sheffield.ac.uk (S.P.A.)

Contents

Experimental Section S2
Materials S2
Characterization Techniques S2
Synthesis Protocols S5
Supplementary Characterization Data for G_{59} macro-CTA and G_{59}-H_{400} Diblock Copolymer Vesicles .S8
Supplementary Characterization Data for G_{59}-H_{400}-O_x Triblock Copolymer Nano-Objects S10
Kinetic Studies during the Synthesis of G_{59}-H_{400}-O_x Triblock Copolymer Nano-Objects by RAFT-Mediated Aqueous PIDA S14
Thermoresponsive Behavior of G_{59}-H_{400} Diblock Copolymer Vesicles and G_{59}-H_{400}-O_x Triblock Copolymer Nano-Objects S16
References S17

Experimental Section

Materials

4,4′-Azobis(4-cyanovaleric acid) (≥98.0%, ACVA), 4-cyano-4-(phenylcarbonothioylthio)pentanoic acid (>97%, CPAD) and lithium bromide (≥99%, LiBr) were purchased from Sigma-Aldrich UK and were used without further purification. Oligo(ethylene glycol) methyl ether methacrylate (average M_n = 300, OEGMA) was also purchased from Sigma-Aldrich and passed through a column of basic alumina (VWR Chemicals, UK) to remove inhibitor prior to use. Glycerol monomethacrylate (GMA) and 2-hydroxypropyl methacrylate (HPMA; mixture of 75 mol% 2-hydroxypropyl methacrylate and 25 mol% 2-hydroxyisopropyl methacrylate) were kindly donated by GEO Specialty Chemicals, UK and were used without further purification. 2,2'-Azobis[2-(2-imidazolin-2-yl)propane]dihydrochloride (97%, VA-044) was purchased from Wako Chemicals, Japan. Methanol-d_4 (99,8%, CD_3OD) was purchased from Goss Scientific Instruments Ltd, UK. All other solvents were purchased from Fisher Scientific, UK and were used as received. Deionized water (pH 6.8) was obtained using an Elga Elgastat Oprion 3A water purification system. Copper/palladium and Quantifoil® holey carbon-coated copper grids were purchased from Agar Scientific, UK.

Characterization Techniques

NMR Spectroscopy. ^{1}H NMR spectra (CD_3OD) were recorded at 400 MHz using a Bruker Ascend™ 400 spectrometer. Chemical proton shifts are reported as δ in parts per million (ppm) and are expressed relative to the residual solvent peak (CH_3OD, δ = 3.32 ppm).

Size Exclusion Chromatography. Size exclusion chromatography (SEC) analysis was performed using an Agilent 1260 Infinity GPC system equipped with an Agilent guard column (PLgel 5 μm) and two Agilent Mixed-C columns (PLgel 5 μm). Detection was conducted using a differential refractive index (RI) and a UV-visible detector set to λ = 305 nm. The mobile phase was DMF (HPLC grade) containing 10 mM LiBr at 60 °C at flow rate of 1.0 mL min^{-1}. Number-average molecular weights (M_n), weight-average molecular weights (M_w) and dispersities ($Đ = M_w/M_n$) were calculated using a series of near-monodisperse poly(methyl methacrylate) (PMMA) calibration standards.

Dynamic Light Scattering. Dynamic light scattering (DLS) analysis was performed using a Malvern Zetasizer Nano ZS instrument equipped with a 4 mW He–Ne 633 nm laser and an avalanche photodiode detector. Back-scattered light was detected at an angle of 173° and measurements were

conducted at a copolymer concentration of 0.1% w/w at either 25 °C or 5 °C. Malvern Zetasizer Software v7.11 was used to calculate hydrodynamic diameters (D_h) *via* the Stokes-Einstein equation, which assumes perfectly monodisperse, non-interacting spherical particles. Data were averaged over at least three consecutive runs with at least ten measurements being recorded for each run.

Aqueous Electrophoresis. Zeta potentials were determined *via* aqueous microelectrophoresis using the same Malvern Zetasizer Nano ZS instrument at 25 °C. Measurements were conducted at a copolymer concentration of 0.1% w/w at pH = 6.8 and data were averaged over at least three consecutive runs with at least ten measurements being recorded for each run. In each case, zeta potential was calculated from the corresponding electrophoretic mobilities (μ_E) by using the Henry's correction for the Smoluchowski equation ($\mu_E = 4\pi\ \varepsilon_0\ \varepsilon_r\ \zeta\ (1+\kappa r)/6\pi\ \mu$).

Transmission Electron Microscopy. Dry-state stained transmission electron microscopy (TEM) images were recorded using a Philips CM100 microscope equipped with a Gatan 1k CCD camera and operating at an acceleration voltage of 100 kV. One 8 μL droplet of a 0.1% w/w aqueous copolymer dispersion was deposited *via* micropipette onto a plasma-treated carbon-coated copper/palladium grid at either 25 °C or 5 °C. After roughly 1 min, excess sample was blotted from each grid using filter paper and the grid was subsequently stained by depositing an 8 μL droplet of a 0.75% w/w aqueous solution of uranyl formate for 1 min prior to blotting and drying under vacuum.

Cryogenic transmission electron microscopy (cryo-TEM) imaging was performed using an FEI Tecnai Arctica microscope operating at an acceleration voltage of 200 kV. Cryo-TEM samples were prepared by depositing 8 μL of a 1.0% w/w aqueous copolymer dispersion onto a plasma-treated Quantifoil® holey carbon-coated copper grid, followed by blotting for approximately 4 s and then plunging into a pool of liquid ethane to vitrify the sample using a Leica EM GP automatic plunge freezer (25 °C, 99% humidity). Transfer of the vitrified grids into a pre-cooled cryo-TEM holder was performed at −196 °C prior to microscopic analysis. Mean membrane thicknesses for G_{59}-H_{400} diblock copolymer vesicles were determined by analyzing at least 100 particles in each case.

Small-Angle X-Ray Scattering. SAXS patterns were collected using a Xenocs Xeuss 2.0 laboratory beamline equipped with a 2D Dectris Pilatus 1M detector, two sets of scatterless slits for collimation and an Excillum liquid gallium MetalJet X-ray source (λ = 1.34 Å). Aqueous copolymer dispersions at 1.0% w/w were placed in a glass capillary (2.0 mm diameter) and the sample temperature was controlled using a HFSX350-CAP heating/cooling capillary holding stage (Linkam Scientific Instruments Ltd, Tadworth, UK). SAXS data were recorded over a scattering vector range of 0.004 Å^{-1} < q < 0.100 Å^{-1}, where $q = 4\pi \sin\theta/\lambda$ and θ is one-half of the scattering angle. One-dimensional (1D)

scattering curves were obtained by azimuthal binning and averaging of the corresponding two-dimensional scattering patterns using software packages supplied by the instrument manufacturer. Calibration, background subtraction, normalization and further analysis of the 1D data were performed using Irena SAS macros for Igor Pro.[1]

Rheology. Rheology measurements were performed using a TA Instruments AR-G2 rheometer equipped with a variable-temperature Peltier plate and a 40 mL 2° aluminum cone. The complex viscosity ($|\eta^*|$), storage modulus (G') and loss modulus (G'') of aqueous copolymer dispersions were measured as a function of applied strain and solution temperature. Aqueous copolymer dispersions at 10-11% w/w were equilibrated at each temperature for 10 min prior to analysis. Percentage strain sweeps from 0.1 to 20% were conducted at either 25 °C or 5 °C using a constant angular frequency of 1.0 rad s^{-1}.

Synthesis Protocols

Synthesis of poly(glycerol monomethacrylate)$_{59}$ (G_{59}) macro-CTA *via* RAFT solution polymerization

The RAFT solution polymerization of GMA was conducted according to previously reported experimental protocols.[2, 3] Briefly, GMA (30.0 g, 187 mmol, 70 eq), CPAD (0.748 g, 2.68 mmol, 1.0 eq) and ACVA (0.150 g, 0.54 mmol, 0.20 eq; [CPAD]/[ACVA] molar ratio = 5.0) were dissolved in anhydrous ethanol (30.9 g, 50% w/w solids) in a round bottom flask. The flask was then sealed and the resulting reaction mixture was purged with dry N_2(g) for 30 min prior to immersing the flask into a pre-heated oil bath at 70 °C for 180 min. The polymerization was terminated by removing the flask from the oil bath and cooling to 20 °C while exposing the reaction solution to air. A final GMA conversion of 85% was determined by ^{1}H NMR analysis in CD_3OD. The resulting G_{59} macro-CTA was isolated by precipitation into a ten-fold excess of dichloromethane, followed by filtration and drying under vacuum to yield the final pure polymer as a pink powder. End-group analysis by ^{1}H NMR spectroscopy (400 MHz, CD_3OD) indicated a mean degree of polymerization (DP) of 59 for the obtained poly(glycerol monomethacrylate) macro-CTA. SEC analysis (DMF + 10 mM LiBr) using a refractive index detector and a series of PMMA calibration standards indicated an M_n of 16.2 kg mol^{-1} and an M_w/M_n of 1.14.

Synthesis of poly(glycerol monomethacrylate)$_{59}$-poly(2-hydroxypropyl methacrylate)$_{400}$ (G_{59}-H_{400}) diblock copolymer vesicles by RAFT aqueous dispersion polymerization

A typical experimental procedure employed for the synthesis of G_{59}-H_{400} diblock copolymer vesicles at 20% w/w solids by RAFT aqueous dispersion polymerization was conducted as follows. HPMA (0.656 g, 4.55 mmol, 400 eq), G_{59} macro-CTA (0.110 g, 11.37 μmol, 1.0 eq) and VA-044 (1.21 mg, 3.75 μmol, 0.33 eq – 10 μL aliquot from a 10× concentrated solution; [G_{59} macro-CTA]/[VA-044] molar ratio = 3.0) were placed in a glass vial equipped with a magnetic stirrer bar and dispersed in deionized water (3.07 mL, 20% w/w solids). The vial was then sealed, purged with dry N_2(g) for 20 min and immersed into a pre-heated oil bath at 37 °C for 16 h to ensure full monomer conversion. After this period, the polymerization was terminated by removing the vial from the oil bath and cooling to 20 °C while exposing the reaction solution to air. The final HPMA conversion was >99%, as judged by ^{1}H NMR spectroscopy (400 MHz, CD_3OD). Similar procedures were also adopted for the synthesis of G_{59}-H_{400} diblock copolymer vesicles at either 10% w/w or 15% w/w solids by appropriately adjusting the amount of deionized water required in each case.

Synthesis of poly(glycerol monomethacrylate)$_{59}$-poly(2-hydroxypropyl methacrylate)$_{400}$-poly(oligo((ethylene glycol) methyl ether methacrylate))$_x$ (G$_{59}$-H$_{400}$-O$_x$) triblock copolymer nano-objects by RAFT-mediated aqueous polymerization-induced disassembly (PIDA)

A typical experimental procedure employed for the synthesis of G$_{59}$-H$_{400}$-O$_{200}$ triblock copolymer worms at 11% w/w solids by RAFT-mediated aqueous PIDA is described. G$_{59}$-H$_{400}$ diblock copolymer precursor vesicles (1.0 mL of a 10% w/w copolymer dispersion, 0.10 g, 1.49 µmol, 1.0 eq) and VA-044 (0.16 mg, 0.49 µmol, 0.33 eq – 5 µL aliquot from a 20× concentrated solution; [G$_{59}$-H$_{400}$]/[VA-044] molar ratio = 3.0) were added to a glass vial equipped with a magnetic stirrer bar. In a separate vial, OEGMA (0.089 g, 0.30 mmol, 200 eq) was dissolved in deionized water (0.60 mL) and the resulting solution was transferred to the first vial to yield an 11% w/w aqueous dispersion. The vial containing the reaction mixture was then sealed, purged with dry N_2(g) for 20 min and immersed into a pre-heated oil bath at 37 °C for 16 h to ensure full monomer conversion. After this period, the polymerization was terminated by removing the vial from the oil bath and cooling to 20 °C while exposing the reaction solution to air. Similar procedures were also adopted for the synthesis of G$_{59}$-H$_{400}$-O$_x$ triblock copolymer nano-objects (where x = 35, 70, 100, 200, and 350) at either 15% w/w or 20% w/w solids. In each case, the final OEGMA conversion calculated by ^{1}H NMR spectroscopy (400 MHz, CD_3OD).

Kinetic studies during the synthesis of G$_{59}$-H$_{400}$-O$_x$ triblock copolymer nano-objects by RAFT-mediated aqueous PIDA

The experimental protocol employed for kinetic studies of the synthesis of G$_{59}$-H$_{400}$-O$_{350}$ triblock copolymer spheres at 15% w/w solids by RAFT-mediated aqueous PIDA is described. G$_{59}$-H$_{400}$ diblock copolymer precursor vesicles (6.0 mL of a 15% w/w copolymer dispersion, 0.90 g, 13.4 µmol, 1 eq) and VA-044 (1.43 mg, 4.41 µmol, 0.33 eq – 10 µL aliquot from a 10× concentrated solution; [G$_{59}$-H$_{400}$]/[VA-044] molar ratio = 3.0) were added to a glass vial equipped with a magnetic stirrer bar. In a separate vial, OEGMA (1.40 g, 4.68 mmol, 350 eq) was dissolved in deionized water (7.90 mL) and the resulting solution was transferred to the first vial to yield a 15% w/w aqueous dispersion. The vial containing the reaction mixture was then sealed, purged with dry N_2(g) for 30 min and immersed into a pre-heated oil bath at 37 °C for 16 h. Aliquots (200 µL) were withdrawn from the polymerizing mixture every 30 min for the first 4 h and were diluted immediately after quenching using (a) CD_3OD for ^{1}H NMR spectroscopic analysis to calculate the OEGMA conversion, (b) DMF + 10 mM LiBr for SEC analysis, or (c) DI water for TEM imaging. After 16 h, the polymerization was terminated by

removing the vial from the oil bath and cooling to 20 °C while exposing the reaction solution to air prior to characterization of the aqueous copolymer dispersion by ^{1}H NMR, SEC and TEM.

Temperature-dependent studies of G_{59}-H_{400} diblock copolymer vesicles and G_{59}-H_{400}-O_x triblock copolymer nano-objects

To assess the thermoresponsive behavior of G_{59}-H_{400} diblock copolymer vesicles and G_{59}-H_{400}-$O_{35/200}$ triblock copolymer nano-objects, a 10% w/w aqueous dispersion of G_{59}-H_{400} vesicles or an 11% w/w aqueous dispersion of G_{59}-H_{400}-$O_{35/200}$ nano-objects were first incubated for 24 h at either 25 °C or 5 °C prior to their characterization by DLS, TEM, SAXS and rheology at either temperature.

Supplementary Characterization Data for G_{59} macro-CTA and G_{59}-H_{400} Diblock Copolymer Vesicles

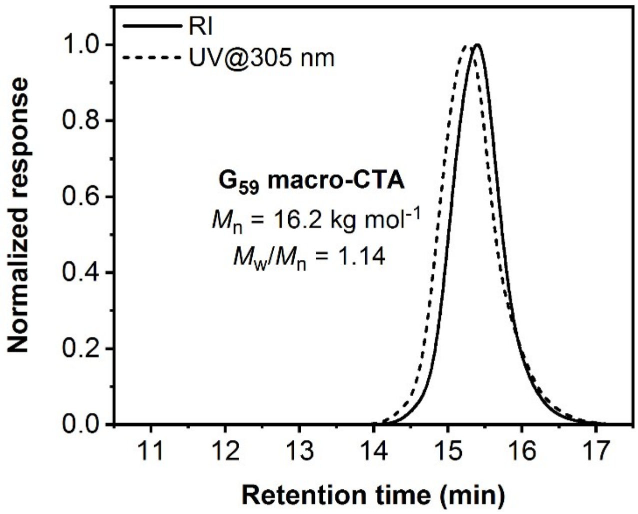

Figure S1. Normalized SEC RI (solid line) and SEC UV (dashed line, λ = 305 nm) traces recorded for the G_{59} macro-CTA, along with corresponding M_n, and M_w/M_n values calculated using a series of PMMA calibration standards (DMF + 10 mM LiBr as the eluent).

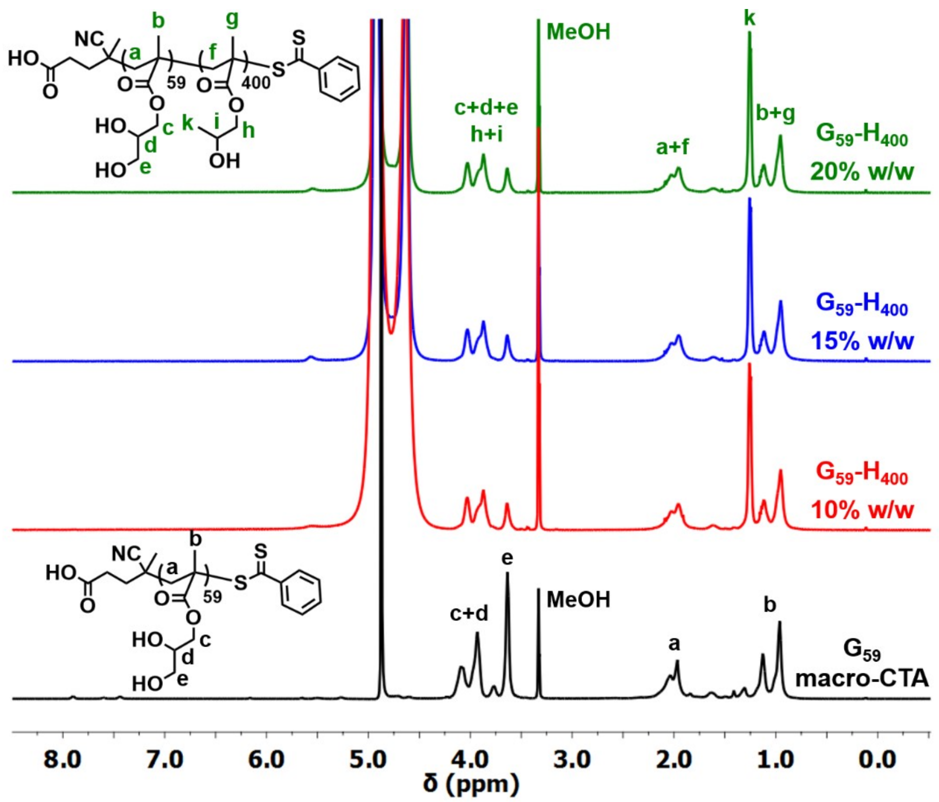

Figure S2. Stacked ^{1}H NMR spectra (CD_3OD) recorded for the purified G_{59} macro-CTA (black line) and molecularly-dissolved G_{59}-H_{400} diblock copolymers prepared *via* RAFT-mediated aqueous PISA at 10% w/w (red line), 15% w/w (blue line) and 20% w/w (green line) solids.

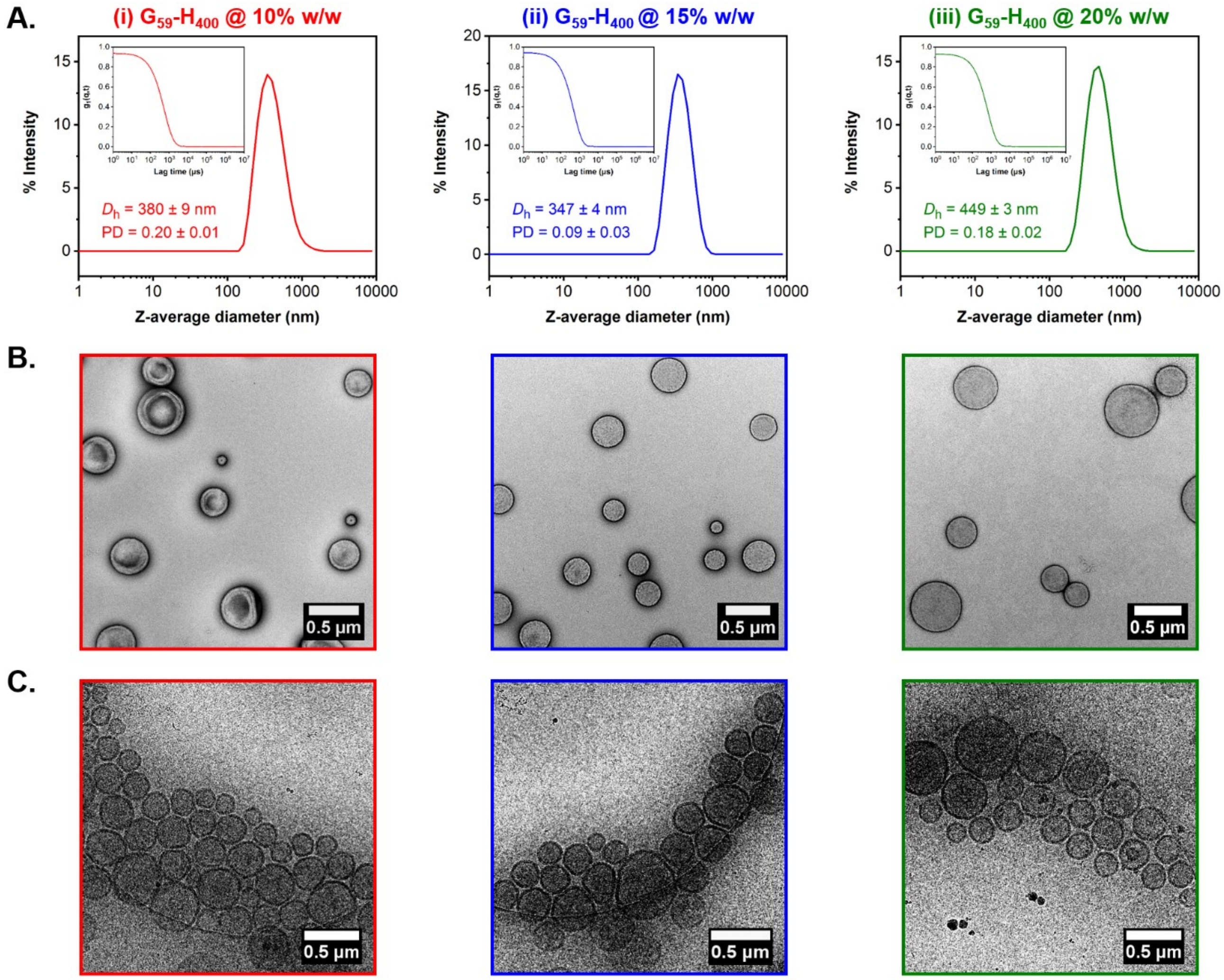

Figure S3. Characterization of G_{59}-H_{400} diblock copolymer precursor vesicles prepared *via* RAFT-mediated aqueous PISA when targeting (i) 10% w/w, (ii) 15% w/w or (iii) 20% w/w solids: (A) Intensity-weighted particle size distributions, mean D_h and PD values measured by DLS (the error indicates the standard deviation from at least three repeat measurements and the inset shows the corresponding autocorrelation function in each case); (B) representative dry-state TEM images, stained using a 0.75% w/w uranyl formate solution; (C) representative cryo-TEM images.

Supplementary Characterization Data for G_{59}-H_{400}-O_x Triblock Copolymer Nano-Objects

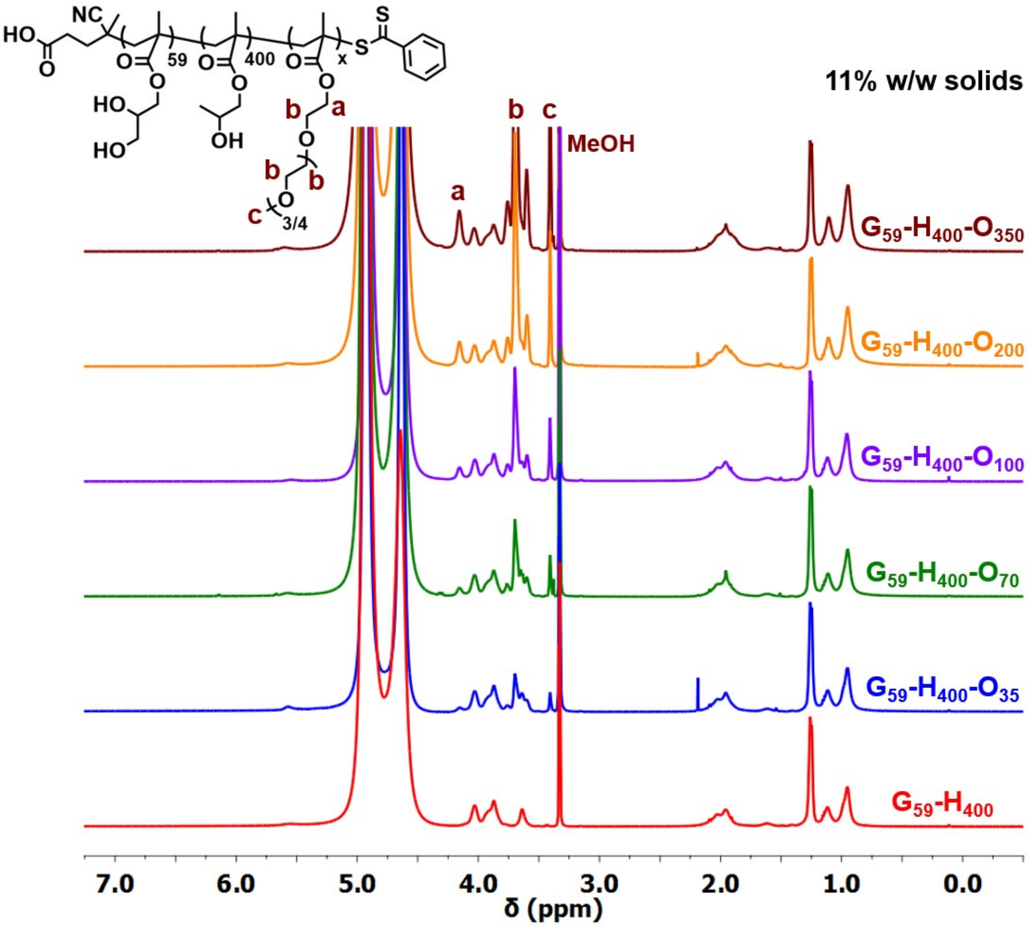

Figure S4. Stacked ^{1}H NMR spectra (CD_3OD) recorded for a series of molecularly-dissolved G_{59}-H_{400}-O_x triblock copolymers (where x = 35, 70, 100, 200 or 350) prepared *via* RAFT-mediated aqueous PIDA at 11% w/w solids.

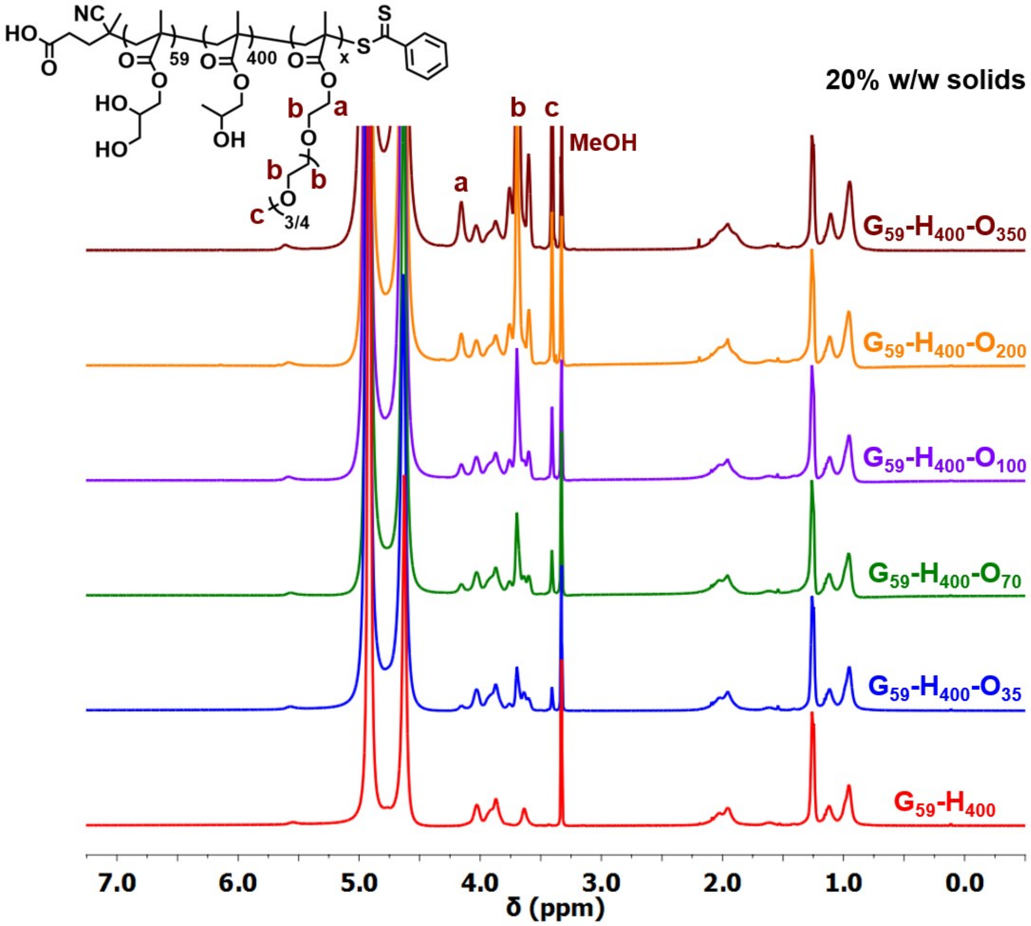

Figure S5. Stacked ^{1}H NMR spectra (CD_3OD) recorded for a series of molecularly-dissolved G_{59}-H_{400}-O_x triblock copolymers (where x = 35, 70, 100, 200 or 350) prepared *via* RAFT-mediated aqueous PIDA at 20% w/w solids.

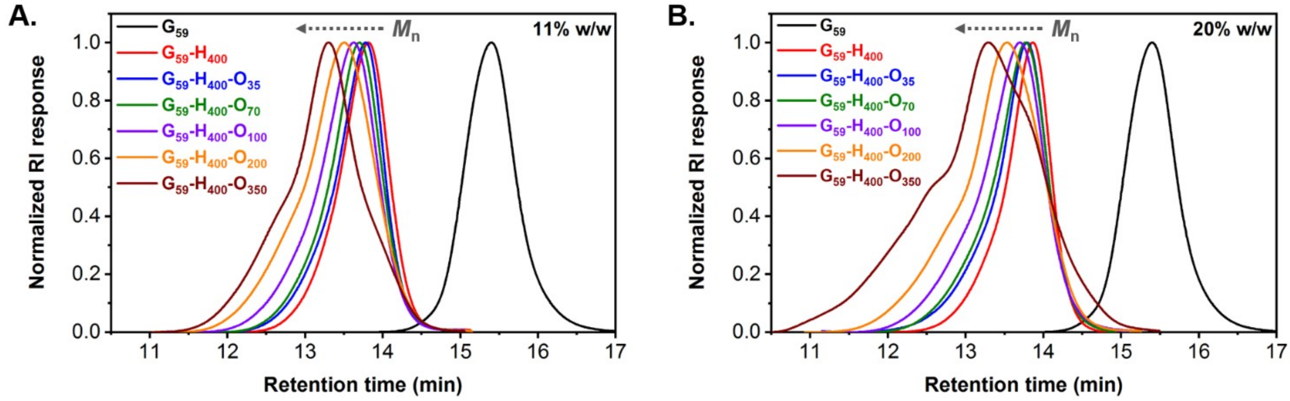

Figure S6. Normalized SEC RI traces recorded for the G_{59} macro-CTA (black line), the G_{59}-H_{400} diblock copolymer precursors (red line) and a series of G_{59}-H_{400}-O_x triblock copolymers (where x = 35, blue line; x = 70, green line; x = 100, purple line; x = 200, orange line; x = 350, burgundy line) at (A) 11% w/w and (B) 20% w/w solids (DMF + 10 mM LiBr as the eluent).

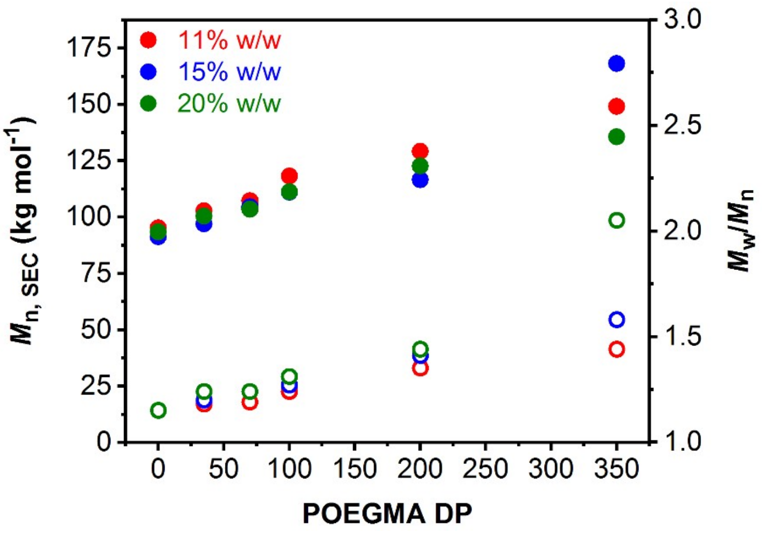

Figure S7. Evolution of M_n (filled circles) and M_w/M_n (empty circles) with increasing target POEGMA DP for a series of G_{59}-H_{400}-O_x triblock copolymers prepared at 11% w/w solids (red circles), 15% w/w solids (blue circles) and 20% w/w solids (green circles), as calculated from SEC RI analysis using a series of PMMA calibration standards (DMF + 10 mM LiBr as the eluent).

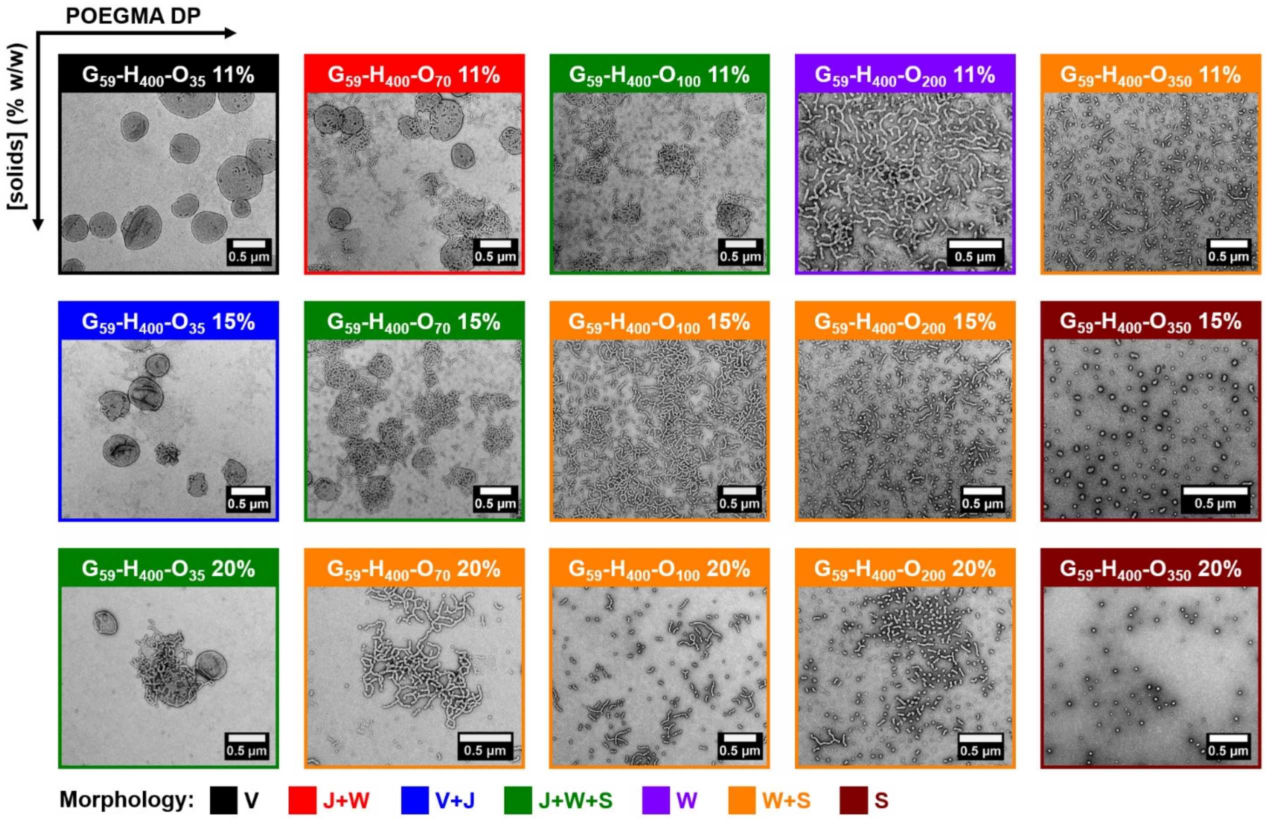

Figure S8. Representative dry-state TEM images recorded for G_{59}-H_{400}-O_x triblock copolymer nano-objects prepared *via* RAFT-mediated aqueous PIDA at 11% w/w, 15% w/w and 20% w/w solids, stained using a 0.75% w/w uranyl formate solution. Each frame color corresponds to a different nano-object morphology (see key).

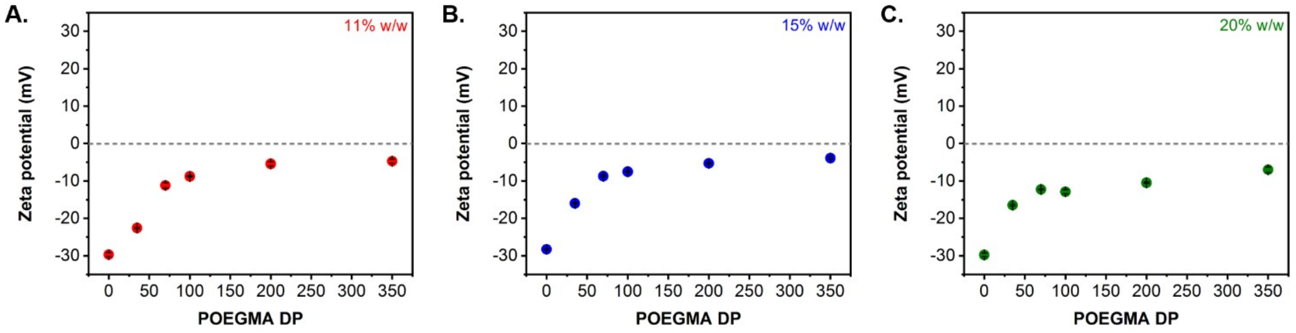

Figure S9. Change in zeta potential on increasing the target POEGMA DP for G_{59}-H_{400}-O_x triblock copolymer nano-objects at (A) 11% w/w solids, (B) 15% w/w solids or (C) 20% w/w solids, as determined by aqueous electrophoresis measurements conducted at pH 6.8.

Table S1. Summary of monomer conversions, SEC data, DLS data, zeta potentials and morphology assignments by dry-state TEM imaging for G_{59}-H_{400} diblock copolymer precursor vesicles and a series of G_{59}-H_{400}-O_x triblock copolymer nano-objects prepared *via* RAFT-mediated aqueous PISA/PIDA.

Copolymer	[solids] (% w/w)	Monomer conversion[a] (%)	$M_{n,\,SEC}$[b] (kg mol^{-1})	M_w/M_n[b]	D_h[c] (nm)	PD[c]	ζ-Potential[d] (mV) @ pH 6.8	Morphology[e]
**G_{59}-H_{400}**	10	>99	95.2	1.15	380 ± 9	0.20 ± 0.01	- 29.7 ± 0.5	V
**G_{59}-H_{400}**	15	>99	91.3	1.15	347 ± 4	0.09 ± 0.03	- 28.3 ± 0.2	V
**G_{59}-H_{400}**	20	>99	93.5	1.15	449 ± 3	0.18 ± 0.02	- 29.8 ± 0.5	V
**G_{59}-H_{400}-O_{35}**	11	>99	102.8	1.18	494 ± 2	0.21 ± 0.01	- 22.6 ± 0.2	V
**G_{59}-H_{400}-O_{70}**	11	95	107.3	1.19	481 ± 11	0.15 ± 0.05	- 11.2 ± 0.7	J + W
**G_{59}-H_{400}-O_{100}**	11	>99	118.3	1.24	378 ± 3	0.32 ± 0.01	- 8.8 ± 0.2	J + W + S
**G_{59}-H_{400}-O_{200}**	11	>99	129.2	1.35	142 ± 3	0.23 ± 0.01	- 5.4 ± 0.6	W
**G_{59}-H_{400}-O_{350}**	11	98	149.2	1.44	190 ± 4	0.34 ± 0.04	- 4.7 ± 0.5	W + S
**G_{59}-H_{400}-O_{35}**	15	>99	97.1	1.20	416 ± 3	0.15 ± 0.01	- 16.0 ± 0.2	V + J
**G_{59}-H_{400}-O_{70}**	15	>99	104.6	1.24	443 ± 10	0.13 ± 0.04	- 8.7 ± 0.2	J + W + S
**G_{59}-H_{400}-O_{100}**	15	>99	111.1	1.27	620 ± 6	0.33 ± 0.01	- 7.5 ± 0.1	W + S
**G_{59}-H_{400}-O_{200}**	15	>99	116.7	1.41	161 ± 1	0.51 ± 0.06	- 5.3 ± 0.2	W + S
**G_{59}-H_{400}-O_{350}**	15	>99	168.3	1.58	130 ± 1	0.22 ± 0.01	- 3.9 ± 0.3	S
**G_{59}-H_{400}-O_{35}**	20	>99	100.5	1.24	549 ± 11	0.25 ± 0.01	- 16.5 ± 0.2	J + W + S
**G_{59}-H_{400}-O_{70}**	20	>99	103.5	1.24	724 ± 4	0.41 ± 0.04	- 12.3 ± 0.1	W + S
**G_{59}-H_{400}-O_{100}**	20	>99	111.3	1.31	457 ± 28	0.75 ± 0.07	- 12.9 ± 0.6	W + S
**G_{59}-H_{400}-O_{200}**	20	98	122.7	1.44	317 ± 102	0.64 ± 0.18	- 10.5 ± 0.2	W + S
**G_{59}-H_{400}-O_{350}**	20	>99	135.6	2.05	114 ± 1	0.25 ± 0.01	- 7.0 ± 0.7	S

[a]Monomer conversion calculated from ^{1}H NMR spectroscopy in CD_3OD. [b]M_n and M_w/M_n data calculated by SEC RI analysis relative to a series of PMMA calibration standards (DMF + 10 mM LiBr as the eluent). [c]D_h and polydispersities determined by DLS analysis. [d]Zeta potentials determined by electrophoresis measurements conducted at pH 6.8. [e]Morphologies assigned on the basis of dry-state TEM images, stained using a 0.75% w/w uranyl formate solution (where S = spherical micelles; W = worms; J = jellyfish; and V = vesicles).

Kinetic Studies during the Synthesis of G_{59}-H_{400}-O_x Triblock Copolymer Nano-Objects by RAFT-Mediated Aqueous PIDA

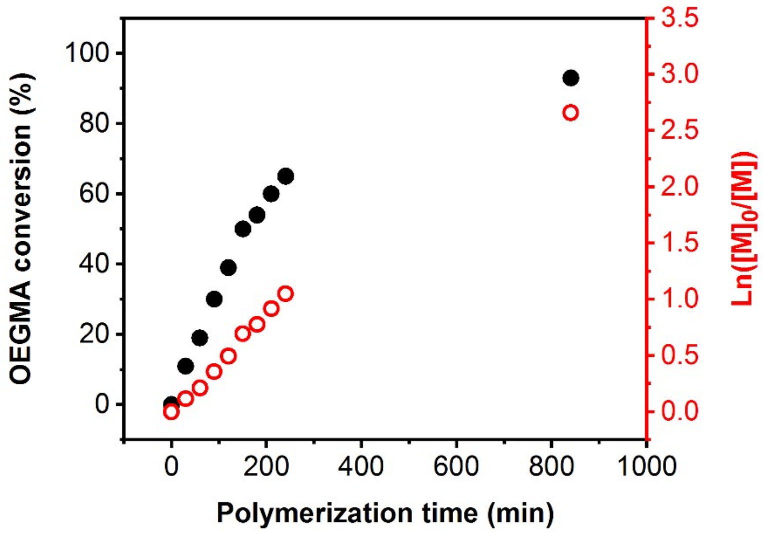

Figure S10. OEGMA conversion *vs*. polymerization time curve (black circles) and corresponding ln([M]$_0$/[M]) *vs*. polymerization time plot (red circles) determined by ^{1}H NMR spectroscopy studies in CD_3OD during the synthesis of G_{59}-H_{400}-O_{350} triblock copolymer spheres *via* RAFT-mediated aqueous PIDA at 37 °C targeting 15% w/w solids.

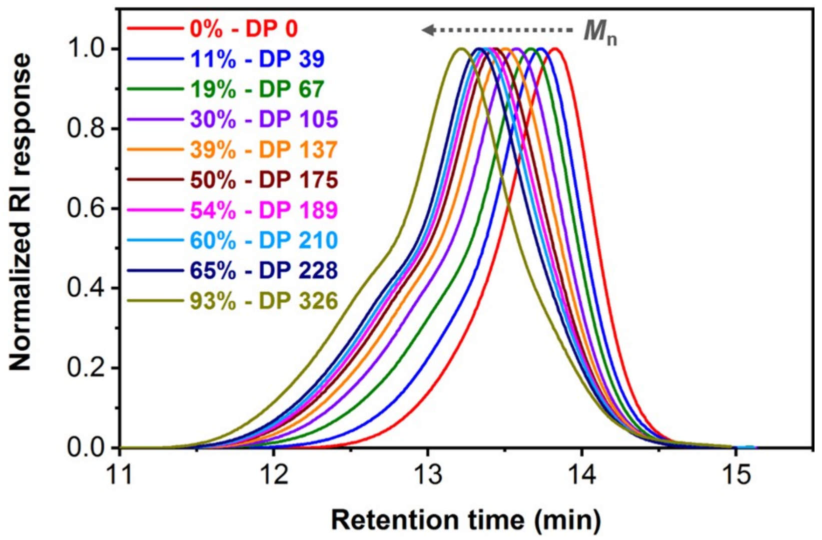

Figure S11. Normalized SEC RI traces recorded at various OEGMA conversions during the synthesis of a G_{59}-H_{400}-O_{350} triblock copolymer *via* RAFT-mediated aqueous PIDA at 37 °C targeting 15% w/w solids (DMF + 10 mM LiBr as the eluent).

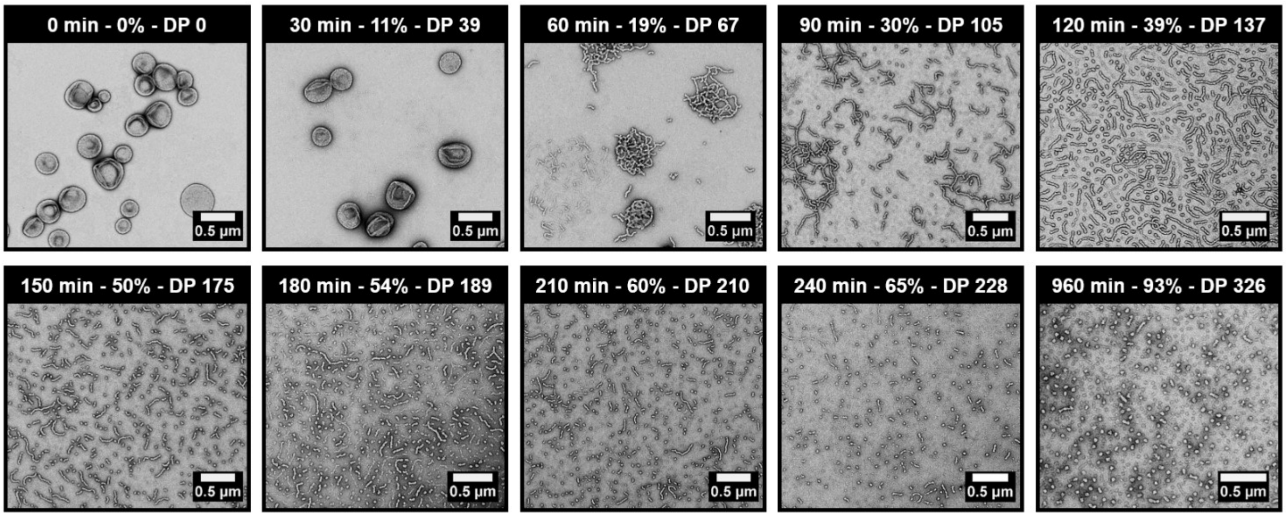

Figure S12. Representative dry-state TEM images recorded for G_{59}-H_{400}-O_x triblock copolymer nano-objects formed during kinetic studies for the synthesis of G_{59}-H_{400}-O_{350} triblock copolymer spheres *via* RAFT-mediated aqueous PIDA at 37 °C targeting 15% w/w solids, stained using a 0.75% w/w uranyl formate solution. The corresponding polymerization time, OEGMA conversion and POEGMA DP are indicated for each image.

Table S2. Summary of monomer conversions, POEGMA DPs, SEC data and morphology assignments by dry-state TEM imaging for G_{59}-H_{400}-O_x triblock copolymer nano-objects formed during kinetic studies for the synthesis of G_{59}-H_{400}-O_{350} triblock copolymer spheres *via* RAFT-mediated aqueous PIDA at 37 °C targeting 15% w/w solids.

**Time (min)**	**OEGMA conversion[a] (%)**	**POEGMA DP[b]**	**$M_{n, SEC}$[c] (kg mol^{-1})**	**M_w/M_n[c]**	**Morphology[d]**
0	0	0	95.8	1.16	V
30	11	39	106.3	1.20	V + J
60	19	67	117.2	1.25	J + W + S
90	30	105	128.4	1.28	W + S
120	39	137	135.7	1.30	W + S
150	50	175	143.4	1.31	W + S
180	54	189	146.8	1.32	W + S
210	60	210	150.3	1.33	W + S
240	65	228	155.3	1.34	W + S
960	93	326	169.5	1.38	S

[a]Monomer conversion calculated from ^1H NMR spectroscopy in CD_3OD. [b]Calculated from the corresponding OEGMA conversion. [c]M_n and M_w/M_n data calculated by SEC RI analysis relative to a series of PMMA calibration standards (DMF + 10 mM LiBr as the eluent). [d]Morphologies assigned on the basis of dry-state TEM images, stained using a 0.75% w/w uranyl formate solution (where S = spherical micelles; W = worms; J = jellyfish; and V = vesicles).

Thermoresponsive Behavior of G_{59}-H_{400} Diblock Copolymer Vesicles and G_{59}-H_{400}-O_x Triblock Copolymer Nano-Objects

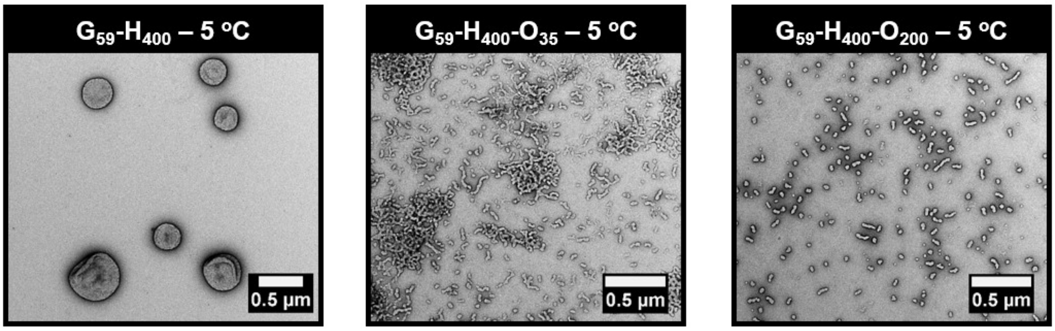

Figure S13. Representative dry-state TEM images recorded for G_{59}-H_{400} diblock copolymer vesicles, G_{59}-H_{400}-O_{35} triblock copolymer worms and G_{59}-H_{400}-O_{200} triblock copolymer spheres (plus a population of short worms) at 5 °C after incubation at this temperature for 24 h prior to imaging, stained using a 0.75% w/w uranyl formate solution.

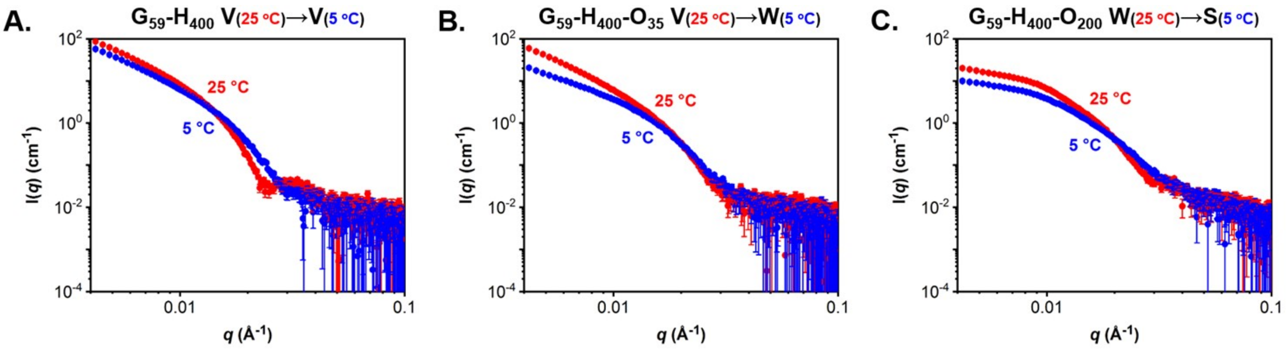

Figure S14. Overlaid SAXS patterns recorded for (A) G_{59}-H_{400} diblock copolymer vesicles, (B) G_{59}-H_{400}-O_{35} triblock copolymer nano-objects, and (C) G_{59}-H_{400}-O_{200} triblock copolymer nano-objects at 25 °C (red circles) and 5 °C (blue circles) after incubation at each temperature for 24 h prior to analysis.

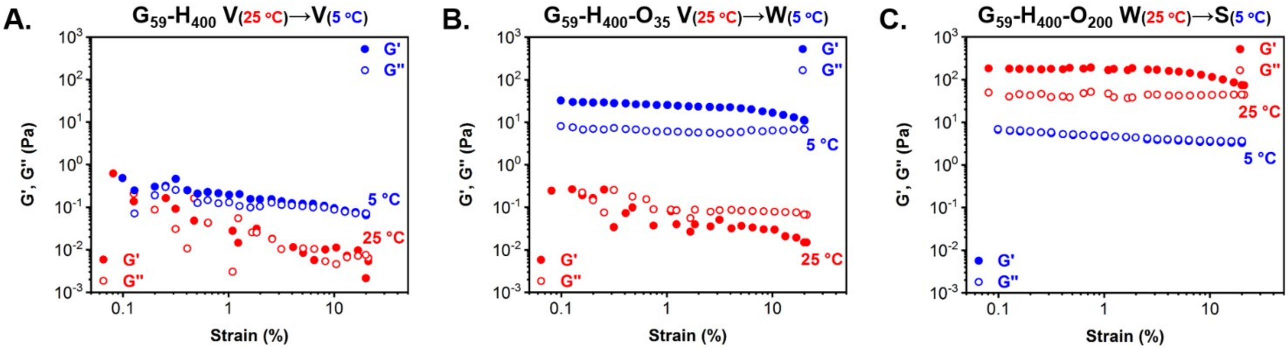

Figure S15. Storage moduli (G′; filled circles) and loss moduli (G″; empty circles) as a function of applied strain percentage for (A) G_{59}-H_{400} diblock copolymer vesicles, (B) G_{59}-H_{400}-O_{35} triblock

copolymer nano-objects, and (C) G_{59}-H_{400}-O_{200} triblock copolymer nano-objects at 25 °C (red circles) and 5 °C (blue circles) after incubation at each temperature for 24 h prior to analysis.

References

1. J. Ilavsky and P. R. Jemian, *J. Appl. Crystallogr.*, 2009, **42**, 347-353.
2. C. J. Mable, R. R. Gibson, S. Prevost, B. E. McKenzie, O. O. Mykhaylyk and S. P. Armes, *J. Am. Chem. Soc.*, 2015, **137**, 16098-16108.
3. N. J. Warren, M. J. Derry, O. O. Mykhaylyk, J. R. Lovett, L. P. D. Ratcliffe, V. Ladmiral, A. Blanazs, L. A. Fielding and S. P. Armes, *Macromolecules*, 2018, **51**, 8357-8371.
